# Suppressing SENP1 inhibits esophageal squamous carcinoma cell growth via SIRT6 SUMOylation

**DOI:** 10.1007/s13402-024-00956-4

**Published:** 2024-07-02

**Authors:** Jianmin Gu, Shaoyuan Zhang, Dong Lin, Wenhan Wang, Jinke Cheng, Quan Zheng, Hao Wang, Lijie Tan

**Affiliations:** 1 Department of Thoracic Surgery, Zhongshan Hospital, Fudan University, Shanghai, 200032 China; 2 Department of Thoracic Surgery, Shanghai General Hospital, Shanghai Jiaotong University School of Medicine, Shanghai, 200080 China; 3 Department of Biochemistry and Molecular Cell Biology, Shanghai Key Laboratory for Tumor Microenvironment and Inflammation, Shanghai Jiao Tong University School of Medicine, Shanghai, 200025 China; 4 Center for Singl-Cell Omics, School of Public Health, Shanghai Jiao Tong University School of Medicine, Shanghai, 200025 China

**Keywords:** Esophageal squamous cell carcinoma, SENP1, Cell cycle, SUMOylation, SIRT6

## Abstract

**Purpose:**

Esophageal squamous cell carcinoma (ESCC) is a prevalent tumor in the gastrointestinal tract, but our understanding of the molecular mechanisms underlying ESCC remains incomplete. Existing studies indicate that SUMO specific peptidase 1 (SENP1) plays a crucial role in the development and progression of various malignant tumors through diverse molecular mechanisms. However, the functional mechanism and clinical implications of SENP1 in the progression of ESCC remain unclear.

**Methods:**

Bulk RNA-Sequencing (RNA-seq) was used to compare potential genes in the esophageal tissues of mice with ESCC to the control group. The up-regulated SENP1 was selected. The protein level of SENP1 in ESCC patient samples was analyzed by immunohistochemistry and western blot. The potential prognostic value of SENP1 on overall survival of ESCC patients was examined using tissue microarray analysis and the Kaplan-Meier method. The biological function was confirmed through in vitro and in vivo knockdown approaches of SENP1. The role of SENP1 in cell cycle progression and apoptosis of ESCC cells was analyzed by flow cytometry and western blot. The downstream signaling pathways regulated by SENP1 were investigated via using RNA-Seq. SENP1-associated proteins were identified through immunoprecipitation. Overexpression of Sirtuin 6 (SIRT6) wildtype and mutant was performed to investigate the regulatory role of SENP1 in ESCC progression in vitro.

**Results:**

Our study discovered that SENP1 was upregulated in ESCC tissues and served as a novel prognostic factor. Moreover, SENP1 enhanced cell proliferation and migration of ESCC cell lines in vitro, as well as promoted tumor growth in vivo. Thymidine kinase 1 (TK1), Geminin (GMNN), cyclin dependent kinase 1(CDK1), and cyclin A2 (CCNA2) were identified as downstream genes of SENP1. Mechanistically, SENP1 deSUMOylated SIRT6 and subsequently inhibited SIRT6-mediated histone 3 lysine 56 (H3K56) deacetylation on those downstream genes. SIRT6 SUMOylation mutant (4KR) rescued the growth inhibition upon SENP1 depletion.

**Conclusions:**

SENP1 promotes the malignant progression of ESCC by inhibiting the deacetylase activity of SIRT6 pathway through deSUMOylation. Our findings suggest that SENP1 may serve as a valuable biomarker for prognosis and a target for therapeutic intervention in ESCC.

**Supplementary Information:**

The online version contains supplementary material available at 10.1007/s13402-024-00956-4.

## Introduction

Esophageal cancer is a highly aggressive gastrointestinal tumor that poses a significant threat to human health. It is the seventh most common cancer worldwide and the sixth leading cause of cancer-related deaths [1]. In China alone, nearly 320,000 new cases were reported in 2020, with around 300,000 deaths, making it the fourth most common cause of cancer deaths [2]. Unfortunately, the clinical efficacy of esophageal cancer treatments is poor due to the hidden symptoms and rapid pathological process, which results in a low 5-year survival rate of only 10–20% [3]. Esophageal cancer can be classified into two types, esophageal squamous cell carcinoma (ESCC) and adenocarcinoma, based on the source of tumor cells. In China, approximately 90% of cases are ESCC [4].

Recently, numerous studies have been conducted to investigate the molecular mechanisms underlying the progression of ESCCs [5, 6]. Researchers have identified several genes closely linked to the development of ESCCs through genomic analysis and other methods. Copy number alterations in the chromosome 11q13.3–13.4 region are often linked to various human diseases, including esophageal squamous carcinoma [4]. Such alterations usually affect the gene expression associated with that specific region. For instance, cyclin D1 (CCDN1), which is located on chromosome 11q13, shows an increased expression as the copy number for that region amplifies [7]. Alterations in specific genes can elicit cascading consequences, as exemplified by the paradigm of the histone deacetylase SIRT6. Genetic mutations within this pivotal tumor suppressor gene can instigate the progression of oncogenesis [8]. Increasing evidence suggested that SIRT6 acts as a tumor suppressor gene, with higher expression levels correlating with improved patient prognosis [9]. Reduced SIRT6 expression was found in multiple cancer cell lines, such as squamous cell carcinoma, hepatocellular carcinoma, colorectal adenocarcinoma, and pancreatic cancer, compared to normal tissue cells [9–12]. Although SIRT6 can be involved in deacetylation, mono-ADP-ribosylation, and defatty-acylation, its tumor suppressor functions primarily depend on its deacetylation activity on histone acetylation [13–15].

SUMO, a ubiquitin-like protein, has been shown to play an important role in regulating the function of multiple proteins. The SUMO protein is conjugated to the target protein through the cascade reaction of E1(activating enzyme)-E2(binding enzyme)-E3 (conjugating enzyme) [16]. SUMOylation is a reversible post-translational modification in eukaryotic cells, and its reversal is catalyzed by SUMO-specific proteases (SENPs) [17]. Nuclear proteins are the group of SUMOylation targets, and these proteins are often involved in gene transcription, DNA damage repair, and other physiological activities [18–20]. It has been reported that SUMOylation plays a critical role in the development of various types of cancer [21]. SENP1 contributes to the progression of prostate and breast cancer via HIF-1α pathways. Moreover, c-MYC and JAK2 were identified as targets of SENP1 in colon cancer, indicating the promising potential of SENP1 for cancer therapy [22–25]. However, the involvement of SUMOylation in ESCC has not been investigated except for one study that showed HSP27 promotes ESCC progression in vitro [26]. Whether SUMOylation regulates ESCC malignancy in vivo remains to be explored. SENP1 is one member of the SENP family and modulates the biological characteristics of certain cancers. However, the role of SENP1 in ESCC has yet to be clarified.

In our study, we analyzed the expression of SENP1 in human ESCC samples and found increased SENP1 mRNA and protein levels in ESCC cells. We observed inhibitory growth and arrested cell cycle when silencing SENP1 in ESCC cell lines. Furthermore, conditional knockout of SENP1 in the esophageal epithelium of mice inhibited the development of ESCC. We also demonstrated that enhanced SUMOylation of SIRT6 mediated the anti-tumor effects induced by SENP1 deficiency in ESCC cells. Additionally, we found that SIRT6 SUMOylation specifically modulates its deacetylation on H3K56.

## Materials and methods

### Main reagents

Paraformaldehyde and puromycin were purchased from Sigma. TriPure Isolation Reagent was purchased from Roche. Chloroform, isopropanol, ethanol, xylene, and neutral resin were purchased from Shanghai Sheng Gong Biological Engineering Co., LTD. Reverse Transcriptase XL and SYBR Premix Ex Taq were purchased from Takara. RPMI 1640 medium was purchased from HyClone. Penicillin-streptomycin and Fetal Bovine Serum were purchased from Gibco. Annexin V-PE-Cy7 (Biolegend, 640950), Propidium Iodide solution (Biolegend, 421301) and 7-AAD Viability Staining Solution (Biolegend, 420403) were purchased from Biolgend. Sodium dodecyl sulfate was purchased from Amresco. Antibodies were purchased from Abcam. Prof. Jinke Cheng (Shanghai Jiao Tong University School of Medicine) kindly gave the Plasmids.

### Main instruments

Leica inverted microscope (Leica), Paraffin sectioning machine (Leica), Conventional PCR instrument (Eppendorf); Fluorescence quantitative PCR instrument LC480 system (Roche); 4 °C high speed freezing centrifuge (Eppendorf); NANODROP 2000 (Thermo); CO_2_ cell incubator (Thermo); Biosafety cabinet (Thermo); Flow cytometry (Becton Dickinson); Vertical swimming apparatus (Shanghai tianneng technology co., LTD.); (Shanghai tianneng technology co., LTD.); Chemiluminescence imaging analyzer (Fujifilm las-4000).

### Patient cohort and specimens

The study included genetically unrelated Chinese Han individuals from China. Patients with ESCC were diagnosed at Zhongshan Hospital, Fudan University, China, between January 2015 and January 2019 and underwent surgery resection. All patients underwent PET-CT before surgery. 270 ESCC tissues without neoadjuvant therapy were collected and prepared into tissue microarrays for further use. The clinical characteristics of all patients were listed in Table 1. TNM staging of ESCC patients was based on the American Joint Committee on Cancer guidelines. This was a retrospective study approved by the Ethics Committee of the Zhongshan Hospital, Fudan University (B2022-271R), and all patients provided signed informed consent for their clinic biological information. TCGA-ESCA data is obtained from UCSC Xena (https://xenabrowser.net/), phenotype data and gene expression were downloaded, normal and esophageal squamous cell carcinoma sample were selected. Finally, we obtained 11 cases of normal tissue samples and 96 cases of ESCC sample data. The detailed protocol for dataset downloading and the final expression data is shown in supplemental text online.

**Table 1:** The clinical characteristics of 270 patients diagnosed with ESCC

Level	SENP1-High (n = 135)	SENP1-Low (n = 135)	*p*-Value
Sex (%)			0.303
Female Male	26 (19.3) 109 (80.7)	33 (24.4) 102 (75.6)	
Age (%)			0.223
≥ 65 < 65	74 (54.8) 61 (45.2)	64 (47.4) 71 (52.6)	
Total lymph node dissection (mean ± SD)	25.8 ± 10.4	24.9 ± 9.1	0.707
pT Stage (%)			<0.001***
Tl-2 T3-4	47 (34.8) 88 (65.2)	99 (73.3) 36 (26.7)	
pN Stage (%)			0.121
N0-1 N2 +	116 (85.9) 19 (14.1)	124 (91.9) 11 (8.1)	
Differentiation (%)			0.296
high/moderate differentiation poor/undifferentiation	88 (65.2) 47 (34.8)	96 (71.1) 39 (28.9)	
SUVmax (%)			<0.001***
> 8 ≤ 8	103 (76.3) 32 (23.7)	76 (56.3) 59 (43.7)	
Surgical procedure (%)			0.013
Ivor Lewis McKeown Sweet	25 (18.5) 90 (66.7) 20 (14.8)	14 (10.4) 111 (82.2) 10 (7.4)	
Ki-67 (%)			<0.001***
Low High	18 (13.3) 117 (86.7)	43 (31.9) 92 (68.1)	
Location (%)			0.924
Up Middle Low EG Junction	15 (11.1) 51 (37.8) 63 (46.7) 6 (4.4)	14 (10.4) 52 (38.5) 65 (48.1) 4 (3.0)	

***P < 0.001 indicates statistical significance

### Animal experiments

C57BL/6 wild-type mice were purchased from Shanghai SLAC Laboratory Animal Co. Ltd. BRL Medicine created the *Senp1* flox/flox mice, while GemPharmatedh Co., Ltd generated the *ED-L2*-Cre mice. The esophageal squamous epithelium-specific knockout mice were obtained from the mating of *Senp1* flox/flox and *ED-L2*-Cre mice. All the mice were kept in a barrier-sustained facility, where they were provided with sterile food and water and kept under specific pathogen-free (SPF) conditions. The use of experimental animals was in accordance with Shanghai Jiaotong University School of Medicine’s regulations. To induce ESCC, six-week-old male mice were given 4-Nitroquinoline N-oxide (Cat. N8141) in drinking water (100 μg/mL) for 16 weeks. After being given regular drinking water for 8 weeks, the mice were euthanized, and esophageal samples were collected.

### Bulk RNA sequencing and data analysis of mice and cell lines

Sangon Biotech (Shanghai) Co., Ltd. conducted RNA-seq analysis of mouse esophageal tissue samples and cell lines. The esophagus of mice was obtained, and the squamous epithelium of the esophagus was isolated and snap-frozen in liquid nitrogen for preservation. RNA was isolated from the samples and cell lines. Then cDNA was prepared using the Geo-seq protocol for sequencing. Sequencing libraries were prepared using the Hieff NGS™ MaxUp Dual-mode mRNA Library Prep Kit for Illumina (YEASEN) and assessed using the Qubit™ ssDNA Assay Kit from Invitrogen™ (Life). Low-quality reads and adaptor sequences were removed by Trim Galore v0.4.4. The clean reads were aligned to mm10 by bowtie2 with default parameter (Langmead and Salzberg, 2012), and uniquely mapping reads were summarized by featureCounts (from Subread package). RNA-seq data were aligned to GRCm39 murine genome and GRCh38 human genome using HISAT2 (version 2.1.0) with default parameters. The raw read counts gene expression matrix underwent processing using the DESeq2. Differentially expressed genes (DEGs) were identified by at least twofold change and a false discovery rate (FDR) adjusted P value of 0.05 by using DESeq2. Bioinformatics analysis was performed using R software (version 4.3.0). The R software used includes “pheatmap” for the heatmap, “ggsci” for color matching and “enrichGO” for GO enrichment analysis.

### Immunohistochemical and tissue microarray analysis

ESCC patient specimens and controls were obtained from Zhongshan Hospital, Fudan University School of Medicine. All patients signed informed consent. The implementation of this project was approved by the ethics committee of Zhongshan Hospital, Fudan University School of Medicine. ESCC specimens were collected from mice with the *Senp1*flox/flox; *Edl2*-Cre genotype. Fresh tissue samples were fixed in 4% paraformaldehyde for 24 h, dehydrated with gradient alcohol and xylene, and embedded in wax blocks. The tissue was cut into 5-micron-thin slices by a paraffin-slicing machine and attached to glass slides. Biopsy in xylene and gradient alcohol fully dewaxing, PBS soak cleaning, with 3% hydrogen peroxide methanol solution to remove the interference of endogenous peroxidase, PBS soak cleaning, in citrate solution at 95 °C for antigen repair, PBS soak cleaning, 10% goat serum closed half an hour at room temperature, removal of sealing fluid, add diluted SENP1 antibody (Abcam, ab108981) or Ki67 antibody(Abcam15580) at 4 °C overnight, PBS soak cleaning, marked biotin drop resistance against rabbit secondary antibody at 37 °C for half an hour, PBS soak cleaning, add biotin coupling of horseradish peroxidase. It was incubated at 37 °C for half an hour, soaked and cleaned by PBS, and colored by DAB with horseradish peroxidase substrates for 2 min, rinsed by tap water, restained by hematoxylin, dehydrated by gradient alcohol and xylene and sealed with neutral resin. Shanghai Runnerbio Technology CO., Ltd. prepared the tissue microarray and conducted the SENP1 staining. We utilized TissueFAXS Plus (version 7.1, TissueGnostics GmbH, Vienna, Austria) image analysis software for image analysis. The total 270 patient specimens were divided into high and low groups based on SENP1 expression in tissue microarrays in a bifurcated manner (n = 135 patients/group).

### Subcutaneous tumorigenesis in athymic nude mice

8-week-old male athymic nude mice were fed in a Specific pathogen-free (SPF) environment with 40–70% humidity at 22 °C for 12 h of light and 12 h of darkness, drinking and eating freely. The nude mice were subcutaneously injected with 5 × 10^6^ shNC cells on the left back and 5 × 10^6^ shSENP1 cells on the right back, respectively. After 3 weeks of feeding, Nude mice were sacrificed after anesthesia, and the tumors on both sides of the back were removed intact. The operation of experimental animals shall be conducted in accordance with the regulations of Shanghai Jiaotong University School of Medicine.

### Cell culture, transfection, and establishment of stable cell lines

The human esophageal squamous cell TE1, TE10, KYSE30, KYSE150, KYSE180, and EC109 was purchased from the cell bank of the Chinese Academy of Sciences and cultured in a constant temperature incubator at 37 °C with 5% CO_2_, using 1640 medium containing 10% fetal bovine serum and 1% Penicillin-streptomycin. Plasmids (Lentiviral expression plasmids were generated by subcloning Sirt6 WT-3XFlag or Sirt6 4KR-3XFlag into pCDH-CMV-MCS-EF1-Puro vector (System Biosciences, Mountain View, CA, USA) and siRNAs (siSIRT6: GGAAGAAUGUGCCAAGUGU) were transfected into ESCC cells by TurboFect Transfection Reagent (Thermo Scientific). Interfering RNA lentivirus of SENP1, SIRT6 and control lentivirus were purchased from VectorBuilder Company (shSENP1#1: ACAAGAAGTGCAGCTTATAAT; shSENP1#2: CTCGATGTCTTAGTTCCAGTA; shSIRT6: GAAGAATGTGCCAAGTGTAAG) and operated according to the virus infection instructions. After 48 h of infection, 1 ug/ml puromycin was added to the medium for screening.

### Cell proliferation, migration, cell cycle, and apoptosis assays

Cell proliferation was measured with Cell Counting Kit-8(Yeasen, Shanghai) every 24 h. Cells were incubated at 37 °C for 2 weeks and stained with crystal violet for colony formation assay.

The monolayer surface was scratched using a sterile 10-μl pipette tip to create wounds. After 24 h, the closure and gaps were observed and captured using an inverted microscope (OLYMPUS). For the migration study, a suspension of single cells in serum-free RPMI-1640 medium (200 µl) was added to the upper chamber of a 24-well plate. In the lower chamber, 600 µl of RPMI-1640 medium with 10% FBS was added to each well. After 24 h, the cells on top of the upper chamber were removed, and the remaining cells were fixed and stained with crystal violet.

Cells were digested by trypsin and centrifuged to collect cell pellets after 24 h of culture respectively. Cell staining was performed using the cell cycle kit (Biolegend) and cell apoptosis kit (Biolegend). The stained cells were filtered by 70 um filter and detected by flow cytometry (Becton Dickinson). Flowjo and Modfit software are used to analyze the results.

### Western blot and immunoprecipitation

Cells were collected and lysed in radioimmunoprecipitation assay (RIPA) buffer (50 mM Tris-HCl, pH 7.4, 400 mM NaCl, 1% Triton X-100, 0.1% SDS, 1 mM PMSF, 10 mM N-ethylmaleimide, and protease inhibitors). Cell lysates were then sonicated and centrifuged at 20,000 × g for 15 min at 4 °C, and the supernatants were added to the appropriate antibody coupled with protein A/G beads. After incubation for 6 h at 4 °C, beads were washed with RIPA buffer, eluted in 2% SDS solution, and then analyzed by immunoblotting. For western blot, cells were lysed with lysates containing 1% SDS and heated at 95 °C for 10 min. Load 10 ul sample onto SDS-PAGE gel. After transfer, incubate membrane in 5% BSA for 1 h at room temperature. Incubate membrane in dilution buffer of SENP1 antibody (Abcam, ab108981), MCL1 (CST, #4572), BCL2 (Abclonal, A19693), Bax (CST, #2772), Caspase-3 (CST, #9661), Thymidine Kinase 1 (CST, #28755), Geminin (CST, #52508), Phospho-CDK1 (Thr161) (CST, #9114), Cyclin A2 (CST, #91500), SIRT6 (Abcam, ab191385), SUMO1 (CST, #4930), H3 (CST, #4499), H3K56Ac (Abclonal, A22565), FLAG (Abcam, ab18230), HA (Abcam, ab9110), ACTIN (Proteintech, 81115-1-RR) with gentle agitation overnight at 4℃. Incubate the membrane with anti-rabbit IgG and HRP-linked antibody for 1 h at room temperature. Images were exposed by Tanon 5200.

### Serum deprivation assay

Cells were starved for 36 h in serum-free medium, then incubated in a complete medium for 8 h prior to collection for cyclin Western blot.

### Data statistics

GraphPad Prism 9.0 software was applied to histogram analysis. The data were expressed as Mean ± standard deviation (Mean ± S.D) and analyzed by Student’s t-test. *p < 0.05 is considered as a statistically significant difference. **p < 0.01 is considered as a highly significant difference. The potential relationship between the expression level of SENP1 and the overall survival of ESCC patients was analyzed using the Kaplan‑Meier method.

## Results

### Elevated expression of SENP1 in human and mouse ESCC samples

**Fig. 1:**
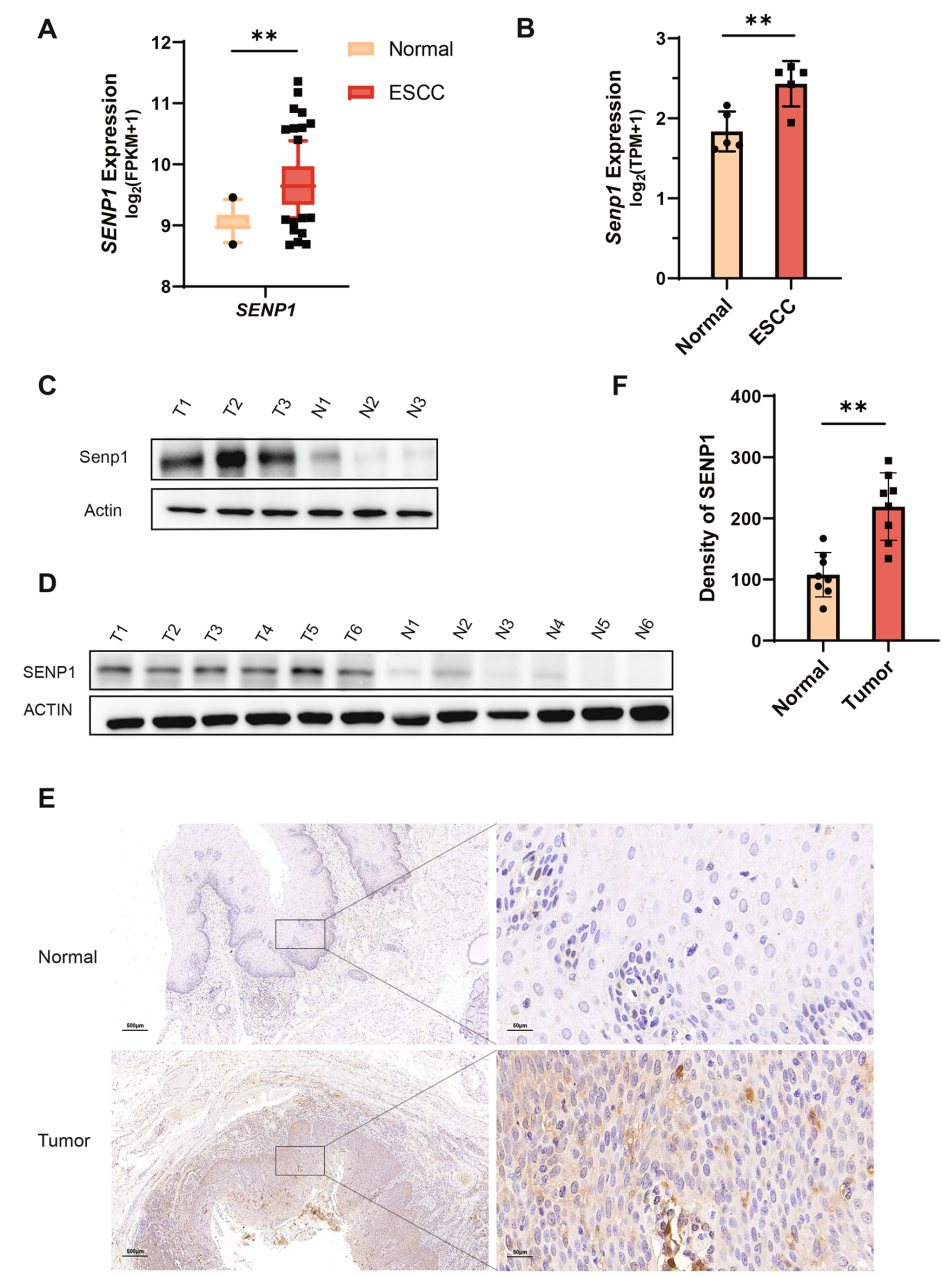
SENP1 is more highly expressed in ESCC than in normal tissue. **A***SENP1* expression in normal esophageal tissues (n = 11) and ESCC (n = 96) from TCGA RNA-seq dataset. **B** Bulk RNA-seq transcriptome analysis showing expression of *Senp1* in mouse esophageal tissues with normal and cancer. Data are mean ± SEM, n = 5 mice/group. **C** Western blot analysis of Senp1 protein levels in mouse normal and ESCC tissues. **D** Western blot analysis of SENP1 protein levels in normal and tumor tissues from patients with ESCC. **E** Representative images of immunohistochemical analysis of SENP1 proteins in normal and tumor tissues. Scale bar = 500 μm. **F** Density of SENP1 in normal and tumor tissues by Image J software (n = 8 patients/group). The significance level was represented by *P < 0.05, **P < 0.01, ***P < 0.001

Oncogenes that promote the development of tumors are often abnormally activated or overexpressed in tumor cells, while tumor suppressor genes are mostly inactivated or deleted in tumor cells. To investigate the role of SENP1 in ESCCs, *SENP1* gene expression from ESCC tissues and normal tissues was analyzed. We used the TCGA-ESCA database to select sequencing results from 96 cases of ESCC and 11 cases of normal tissue based on pathological analysis. The mRNA level of *SENP1* was significantly higher in ESCC tissues than in normal esophageal tissues (Fig. 1A). We then performed bulk RNA-seq on normal epithelial samples obtained from control mice and ESCC samples from 4NQO-treated mice. Transcriptome analysis revealed significantly higher expression of *Senp1* in esophageal tissues of mice with ESCC compared to control group (Fig. 1B). Western blotting also showed increased Senp1 protein levels in esophageal tissues of mice with ESCC compared to control group (Fig. 1C). Additionally, we also found significantly higher expression of SENP1 in the malignant tissues of esophageal cancer patients compared to the paired normal tissues. (Fig. 1D). Furthermore, H&E and immunohistochemical analysis revealed that SENP1 protein expression was significantly higher in the patient’s cancerous tissues compared to their paired normal tissues. (Fig. 1E, F, Supplementary Fig. 1A). These results proved that SENP1 is highly expressed in ESCCs, indicating that SENP1 is likely to play a role in the development of ESCCs.

### SENP1 knockdown inhibited the proliferation and migration of ESCC cells in vitro

**Fig. 2:**
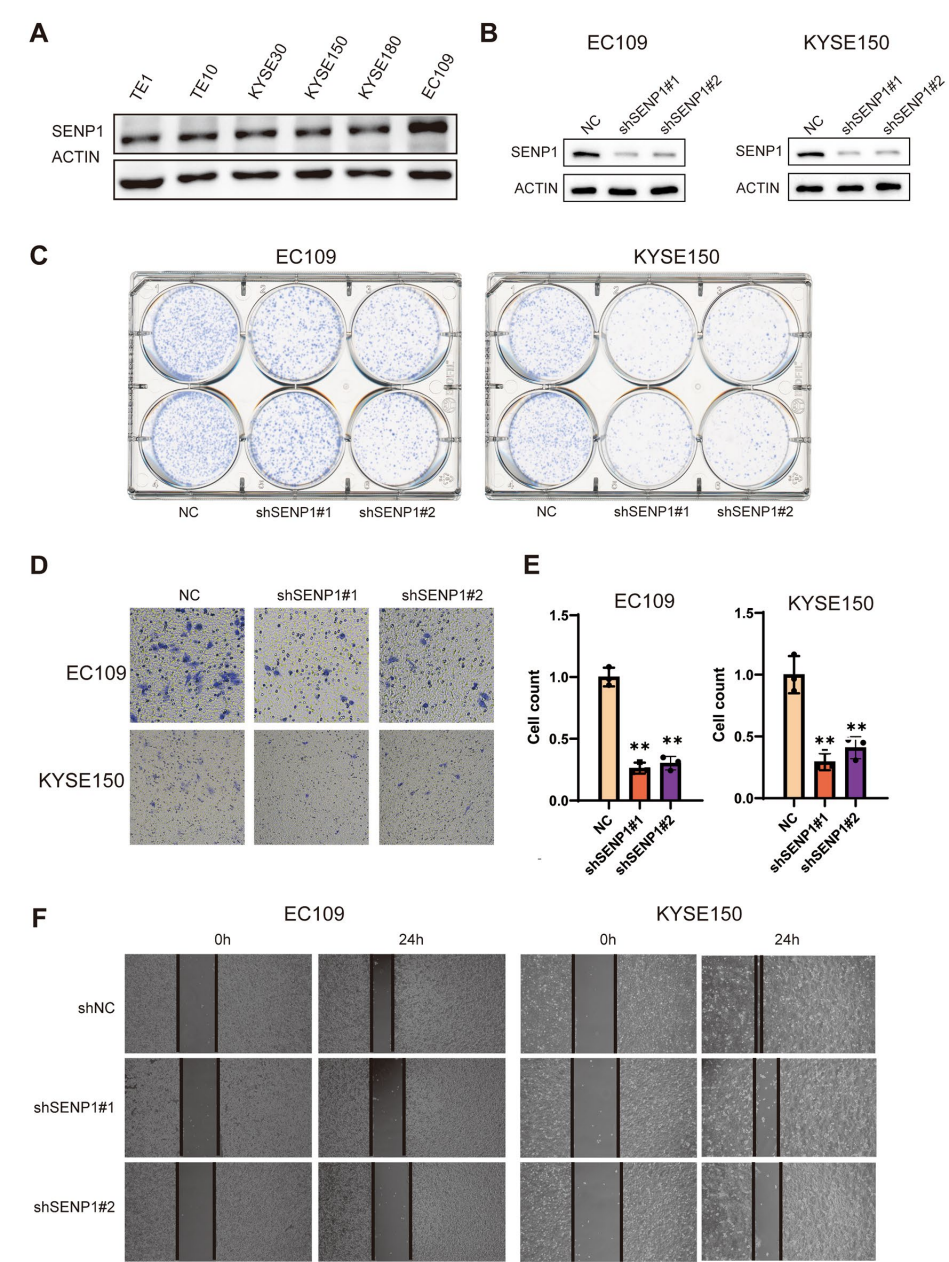
Knocking down SENP1 inhibits the proliferation and migration of ESCC cells. **A** Western blot analysis of SENP1 protein levels in six ESCC cell lines. **B** Western blot analysis of SENP1 protein levels in stably SENP1 knockdown or negative control (NC) EC109 and KYSE150 cells. **C** Colony formation, **D**, **E** Transwell migration, and **F** Would-healing assays were performed in EC109 and KYSE150 with knocking down SENP1. Data are means ± SD from three independent experiments. The significance level was represented by *P < 0.05, **P < 0.01, ***P < 0.001

Since SENP1 was overexpressed in ESCC cells, we speculated whether silence of SENP1 could affect the cell growth of ESCC cells. Firstly, SENP1 expression was analyzed in six ESCC cell lines, including TE1, TE10, KYSE30, KYSE150, KYSE180, and EC109 (Fig. 2A). We found higher levels of SENP1 protein expression in EC109 and KYSE150. EC109 was derived from esophageal cancer patients in China, and KYSE150 was derived from esophageal cancer patients in Japan. These two cell lines are currently the most widely used ESCC cell lines. Stably SENP1 knockdown (KD) in EC109 and KYSE150 cells was established by lentiviral shRNA; we confirmed that SENP1 protein levels were dramatically reduced in shSENP1 cells than in shNC cells (Fig. 2B). We observed that stable knockdown of SENP1 in EC109 and KYSE150 cells led to a significant reduction in cell proliferation in vitro (Fig. 2C). The transwell and wound healing assays demonstrated that SENP1 KD inhibited the invasive and migrative abilities of ESCC cells (Fig. 2D–F)

### SENP1 deficiency inhibits tumor growth in vivo

**Fig. 3:**
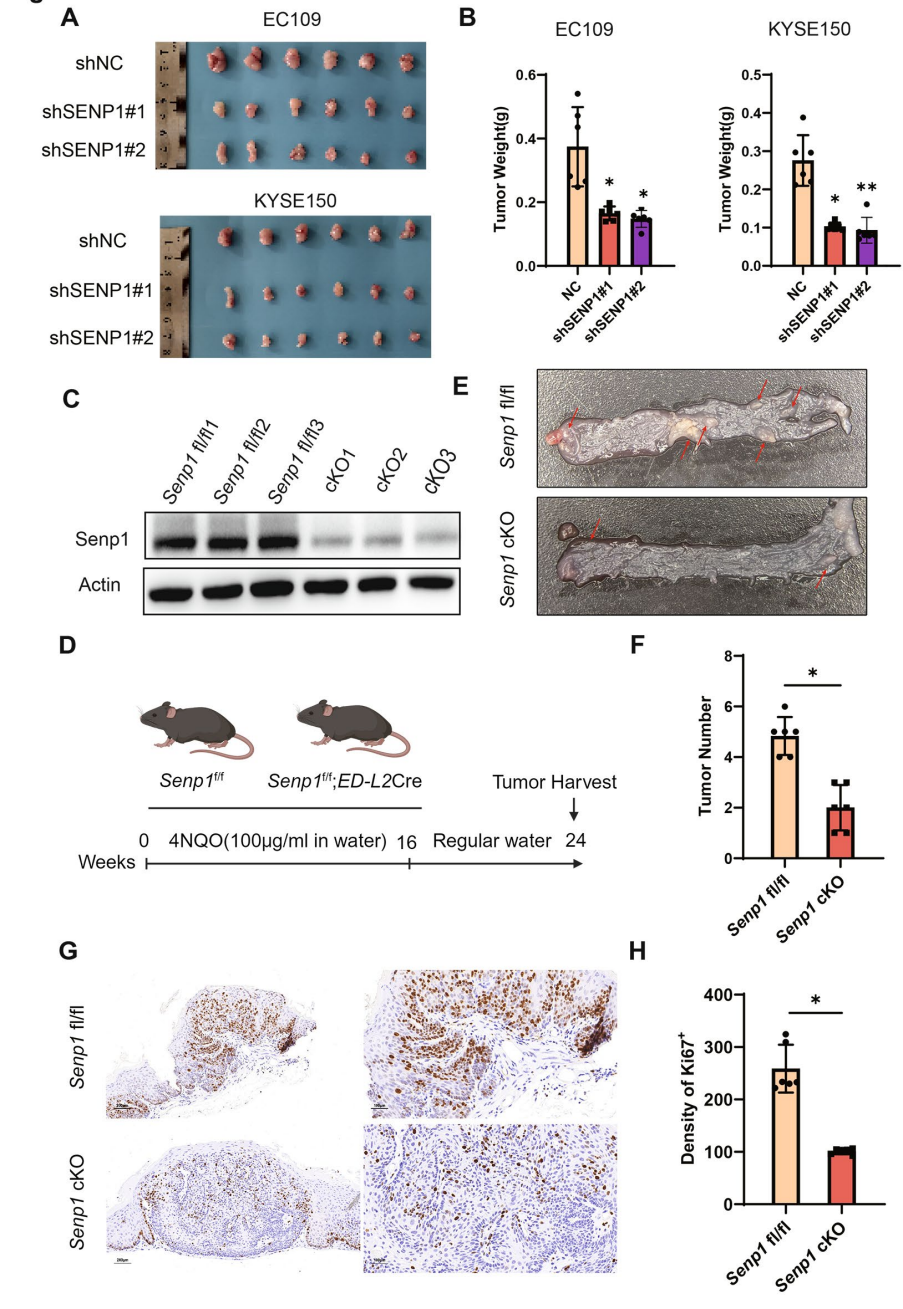
SENP1 deficiency inhibits tumor growth in vivo. **A**, **B** Tumor volume and weight of NC and shSENP1 xenografts in EC109 and KYSE150 are shown (n = 6 mice/group). **C** Western blot indicates Senp1 protein is reduced in cKO Esophageal epithelium. **D** Mice were treated with 4NQO in drinking water (100 μg/ml) for 16 weeks, followed by 8 weeks with regular water. Tumors were harvested at the end of the experiments. **E**, **F*** Senp1* cKO mice had a lower incidence of tumors in their esophagus compared to *Senp1* fl/fl mice. The red arrows in the plot indicate esophageal tumors. The number of tumors per mouse in the esophagus of *Senp1* fl/fl mice is compared to that in cKO mice (n = 6 mice/group). **G*** Senp1* cKO mice showed decreased Ki67 expression in their tumor tissues compared to *Senp1* fl/fl mice. Scale bar: 200 μm (left), 100 μm (right). **H** The percentage of Ki67-positive cells was plotted for both *Senp1* cKO and *Senp1* fl/fl mice (n = 6 mice/group). The significance level was represented by *P < 0.05, **P < 0.01, ***P < 0.001

To investigate the in vivo function of SENP1 in tumor growth, shNC and shSENP1 ESCC cells were subcutaneously injected into the right back of the same athymic nude mice. In vivo experiments showed that transplanted mice with EC109-shSENP1 or KYSE150-shSENP1 had smaller and lighter tumors than control group mice (Fig. 3A, B). Immunohistochemical analysis revealed that Ki67 expression of NC xenografts in EC109 and KYSE150 was higher than of shSENP1 xenografts (Supplementary Fig. 1B, C). Next, we constructed esophageal squamous epithelium specific *Senp1* conditional knockout mice (cKO) by crossing *Senp1* flox/flox and *ED-L2*-Cre mice (Fig. 3C) [27]. We induced esophageal cancer with 4NQO and harvested tissue samples at the age of 24 weeks (Fig. 3D). The number of tumors per mouse was reduced in cKO mice compared to *Senp1* flox/flox mice (Fig. 3E, F). Moreover, H&E and immunohistochemical analysis revealed that the density of Ki67-positive tumor cells was significantly higher in *Senp1* flox/flox mice compared to cKO mice (Fig. 3G, H, Supplementary Fig. 1D). These data suggest that decreased SENP1 expression can inhibit the growth of ESCC cells in vivo.

### Knocking down SENP1 inhibited the cell cycle progression of KYSE150 cells without affecting apoptosis

**Fig. 4:**
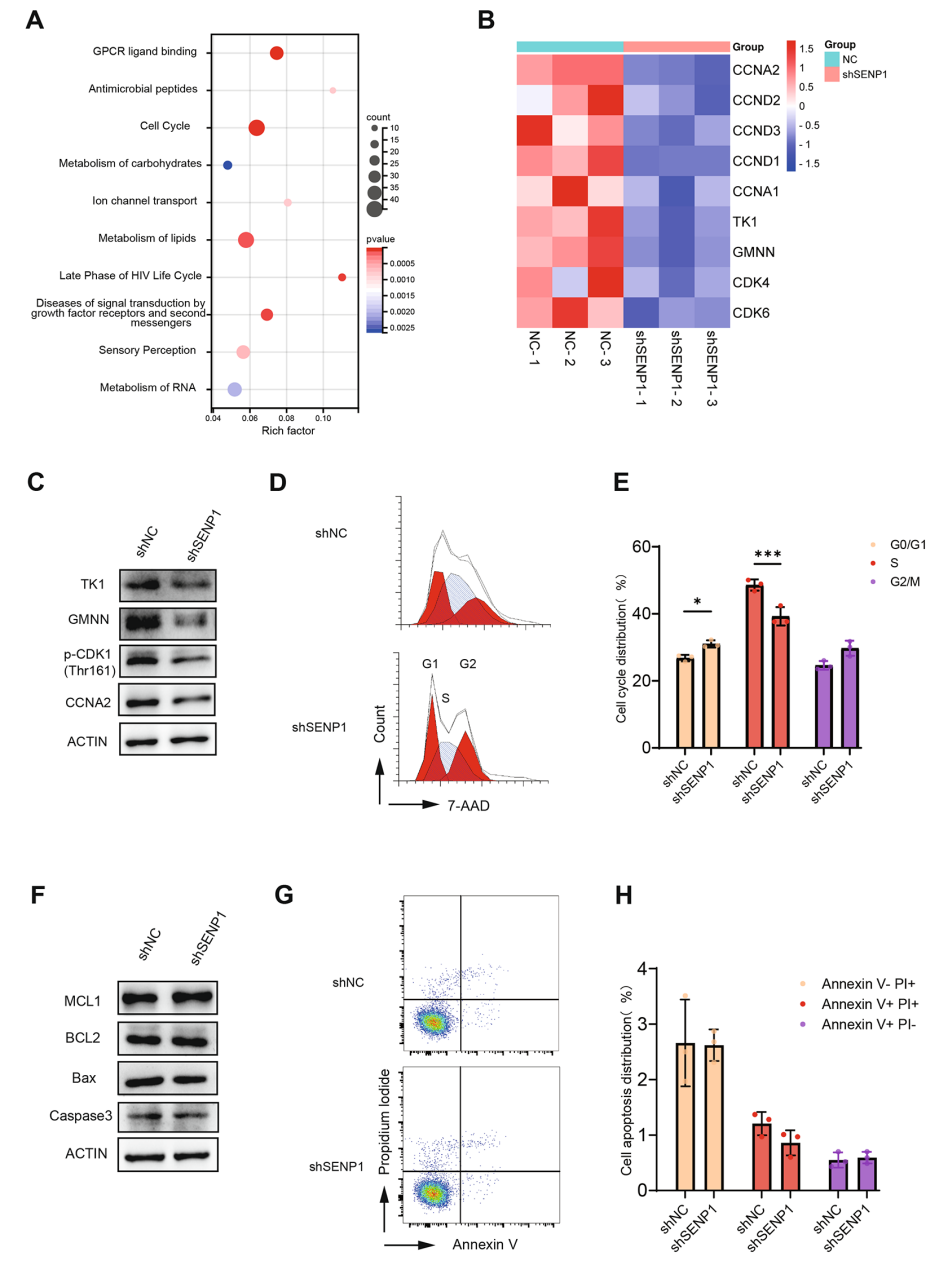
The cell cycle is arrested in SENP1 deficient KYSE150 cells. **A** Gene ontology (GO) analysis of the different genes. **B** Heatmap of representative cell cycle-related differentially expressed genes between shNC and shSENP1 KYSE150 cells. **C** Western blot analysis of TK1, GMNN, p-CDK1, and CCNA2 protein levels after serum deprivation treatment in shNC and shSENP1 KYSE150 cells. **D**, **E** Quantitative measurement of cell cycle phase through flow cytometry following 7-AAD staining. **F** Western blot analysis of MCL1, BCL2, BAX, and Caspase3 protein levels after serum deprivation treatment in shNC and shSENP1 KYSE150 cells. **G**, **H** Quantitative measurement of cell apoptosis through flow cytometry following Annexin V and PI staining. Data are means ± SD from three independent experiments. The significance level was represented by *P < 0.05, **P < 0.01, ***P < 0.001

To gain insights of the mechanism, we conducted bulk RNA-seq to identify dysregulated genes in response to SENP1 deficiency. The Gene Ontology (GO) analysis revealed that genes related cell cycle was significantly altered (Fig. 4A). As shown in the heatmap (Fig. 4B), *Cyclin A* (*CCNA*), *Cyclin D* (*CCND*), *Thymidine Kinase 1* (*TK1*), *Geminin* (*GMNN*) and *Cyclin dependent kinase 1* (*CDK1*), which are responsible for promoting cell proliferation, were all downregulated in shSENP1 cells. To validate this, we induced cell cycle synchronization of KYSE150 cells to the early G1 phase by subjecting them to serum deprivation, followed by this culturing for a duration of 8 h. The expression levels of TK1, GMNN, phosphorylated-CDK1 (p-CDK1) and CCNA2 were decreased in SENP1 KD KYSE150 cells (Fig. 4C). Indeed, inhibiting SENP1 expression caused the cell cycle arrested in the G0/G1 phase and reduced the number of cells in the S and M phases (Fig. 4D, E). On the contrary, we found that the expression of apoptotic marker genes, such as Myeloid cell leukemia sequence 1 (MCL1), B-cell CLL/lymphoma 2 (BCL2), BCL2-associated X protein (Bax), and Caspase 3 (CASP3), were comparable between shNC and shSENP1 cells (Fig. 4F). There was no significant difference in the number of apoptotic cells between the SENP1 KD and control groups (Fig. 4G, H), further indicating that SENP1 didn’t affect KYSE150 cell growth through apoptosis. Taken together, our results declare that the knockdown of SENP1 in ESCC cells slowed down the tumor growth by blocking cell cycle progression and inhibiting cell proliferation without affecting apoptosis.

### SENP1 deletion inhibits ESCC proliferation by enhancing SIRT6-mediated H3K56 deacetylation

**Fig. 5:**
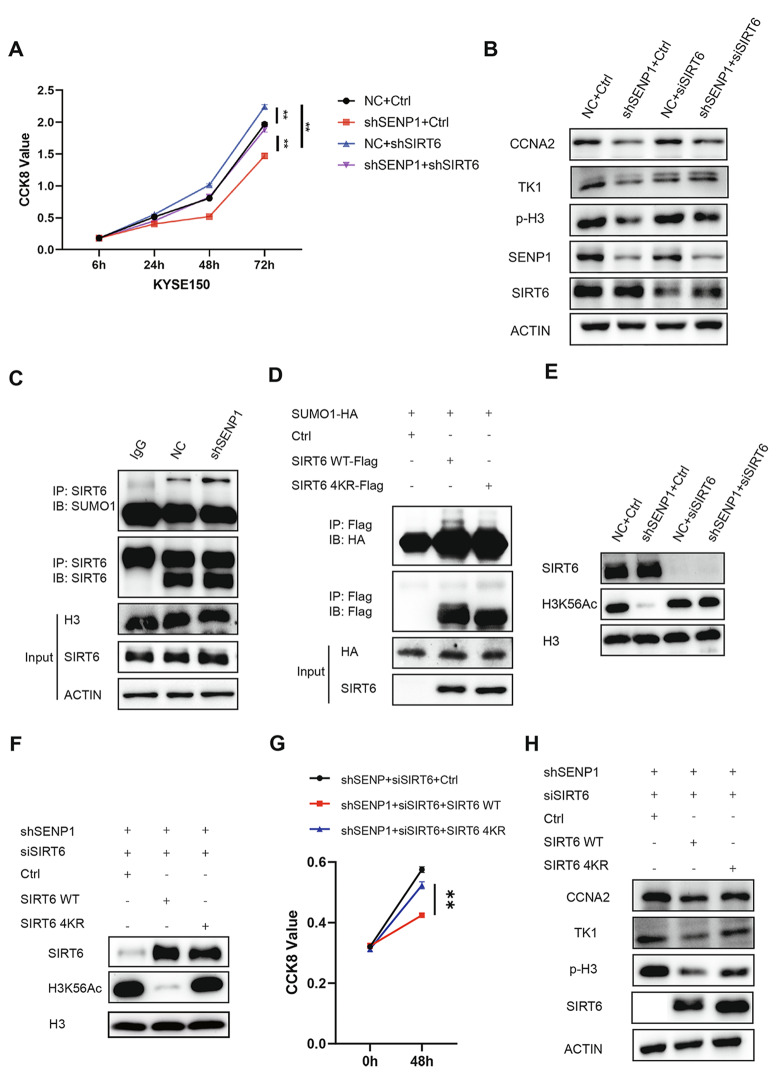
SENP1 regulates the proliferation and cell cycle of KYSE150 cells through the SUMOylation of SIRT6. **A** CCK8 assay was used to detect the cell growth of shNC and shSENP1 KYSE150 cells infected with shSIRT6 lentivirus as indicated. **B** Western blot analysis of CCNA2, TK1, p-H3, SENP1, and SIRT6 protein levels in shNC and shSENP1 KYSE150 cells transfected with siSIRT6. **C** ShNC and shSENP1 KYSE150 cells were immunoprecipitated with anti-SIRT6 antibody and followed by Western blot with anti-SUMO1 or anti-SIRT6 antibody. Whole-cell lysates were blotted with anti-H3 and anti-SIRT6 antibodies. **D** As indicated, KYSE150 cells were transfected with SUMO1-HA, SIRT6-WT-FLAG, or SIRT6-4KR-FLAG plasmids. After 48 hours, the cell lysates were immunoprecipitated with anti-FLAG antibody followed by blotted with anti-HA or anti-FLAG antibody. Whole-cell lysates were blotted with anti-HA and SIRT6 antibodies. **E** Western blot analysis of SIRT6, H3K56Ac, and H3 protein levels in shNC and shSENP1 KYSE150 cells transfected with siRNAs targeting SIRT6. **F** Western blot analysis of SIRT6, H3K56Ac, and H3 protein levels in shSENP1 and siSIRT6 KYSE150 cells transfected with PCDH, SIRT6-WT-FLAG, or SIRT6-4KR-FLAG plasmids as indicated. **G** CCK8 assay was used to detect the cell growth of shSENP1 KYSE150 cells transfected with PCDH, SIRT6-WT-FLAG, or SIRT6-4KR-FLAG plasmids as indicated. **H** Western blot analysis of CCNA2, TK1, p-H3, and SIRT6 protein levels in shSENP1 and siSIRT6 KYSE150 cells transfected with PCDH, SIRT6-WT-FLAG or SIRT6-4KR-FLAG plasmids followed by a serum deprivation treatment as indicated. Data are means ± SD from three independent experiments. The significance level was represented by *P < 0.05, **P < 0.01, ***P < 0.001

SIRT6 has been a well-defined tumor suppressor in multiple cancers [15]. To investigate the role of SENP1 in regulating ESCC cell growth through SIRT6, we first examined the protein expression of SIRT6 in shNC and shSENP1 cells. However, we found no significant difference in SIRT6 protein levels between these two groups (Supplementary Fig. 1E). We then silenced the SIRT6 gene expression in shNC and shSENP1 KYSE150 cells using both shRNA and small interfering RNA (siRNA). The CCK8 assays showed that knockdown of SENP1 expression inhibited cell growth in the KYSE150 cell line. However, this effect of SENP1 deficiency was abolished in shSIRT6 cells (Fig. 5A). As shown in Fig. 5B, the reduced CCNA2, TK1, and phosphorylated-histone 3 (p-H3) proteins in shSENP1 cells could be recovered by knocking down of SIRT6. TK1 increases from the G1 phase, peaks in the S phase, and is subsequently degraded, while CCNA2 peaks in the G2 phase, and Phospho-Histone H3 (Ser10) increases in G2 phase and peaks in M phase and can help chromatin concentration [28–30]. SIRT6 has been discovered to be SUMOylated, which promotes its interaction with c-MYC and suppresses tumorigenesis [31]. To verify whether SENP1 directly targets de-SUMOylation of SIRT6 in ESCC cells, we conducted a co-immunoprecipitation (co-IP) assay and found that SUMO-conjugated SIRT6 was more enriched in shSENP1 than shNC cells (Fig. 5C). We next confirmed that only SIRT6 wild-type (WT) could be conjugated with SUMO proteins but not the mutant with four lysine residues converted to arginine (4KR) in KYSE150 cells (Fig. 5D). Knocking down of SENP1 led to a reduction in H3K56 acetylation levels, while this effect was reversed upon transfection with siSIRT6 (Fig. 5E). Furthermore, the acetylation level of H3K56 in SENP1 and SIRT6 double KD cells could be decreased by overexpression of SIRT6-WT but not SIRT6-4KR (Fig. 5F). The CCK8 assays further showed that ectopic expression of SIRT6-WT but not SIRT6-4KR in SENP1 and SIRT6 double KD cells could inhibit cell growth (Fig. 5G). Consistently, western blotting revealed declines in CCNA2 and TK1 protein levels only in SIRT6-WT but not SIRT6-4KR cells. P-H3 protein levels were reduced in SIRT6-WT, but most protein levels were restored after SIRT6-4KR (Fig. 5H). Collectively, we proved that SIRT6 was essential for SENP1-regulated cell growth in ESCC cells. SUMOylation of SIRT6 acts as a key mediator in the cell growth regulation of SENP1 in ESCC cells.

### SENP1 expression correlates with the pathological stage and prognosis of esophageal cancer patients

**Fig. 6:**
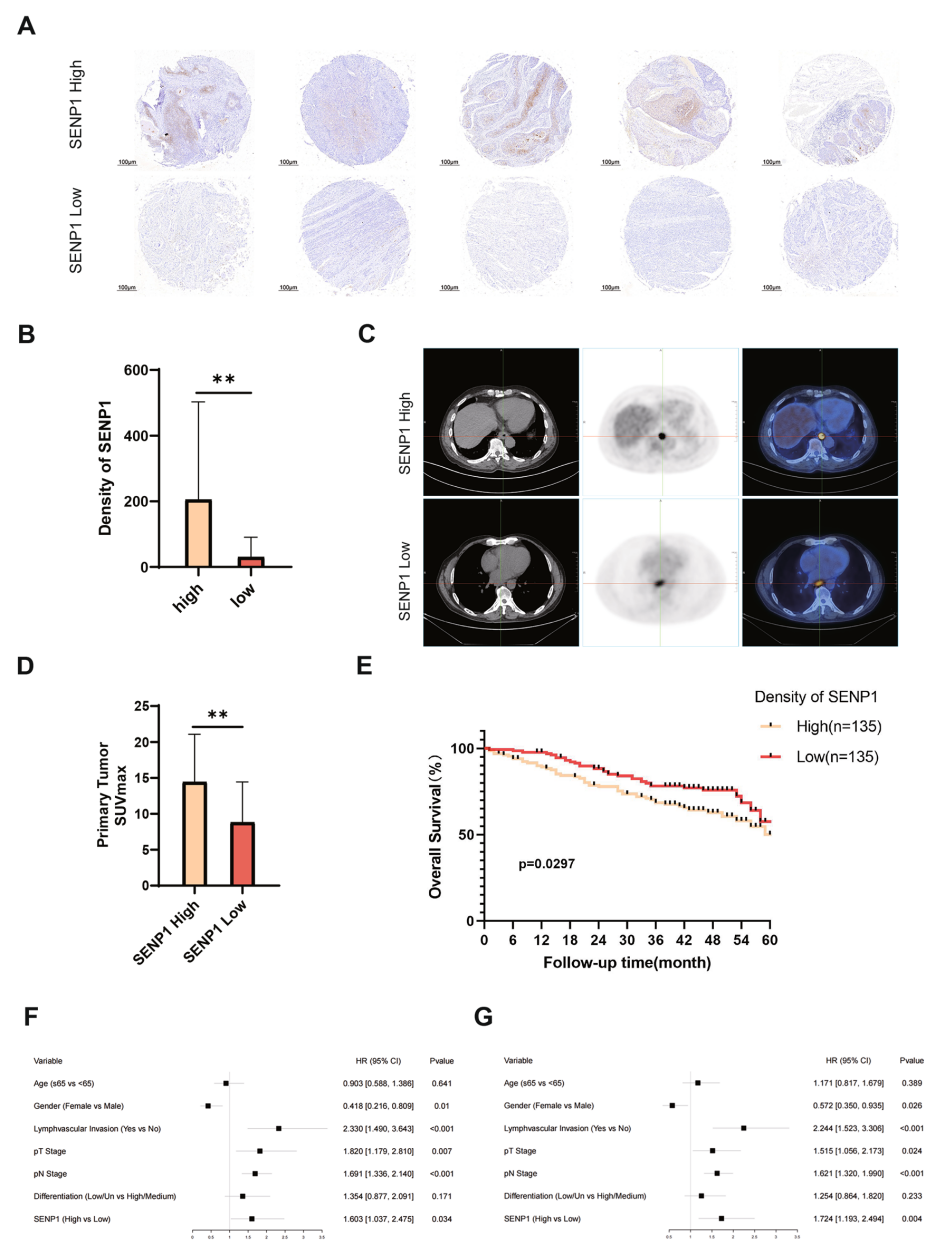
SENP1 expression correlates with the pathological stage and prognosis of esophageal cancer patients. **A** Patients were divided into high and low groups based on SENP1 expression in tissue microarrays in a bifurcated manner (n = 135 patients/group). **B** Density of SENP1 in high and low patients by Image J software. **C**, **D** The SUVmax of PETCT in patients from SENP1 high and low expression groups (n = 135 patients/group). **E** Kaplan-Meier survival curves generated based on the expression of SENP1. **F** OS Cox regression analysis in ESCC patients. **G** RFS Cox regression analysis in ESCC patients. The significance level was represented by *P < 0.05, **P < 0.01, ***P < 0.001

To investigate the correlation between SENP1 and ESCC, the patients were categorized into high and low groups based on the expression of SENP1 in tissue microarrays in a bifurcated manner (as shown in Fig. 6A, B). The characteristics of the study cohort, which consisted of 270 participants, were summarized in Table 1. SENP1 significantly correlated with higher levels of pT Stage and PET-CT SUVmax (Fig. 6C, D), as well as Ki-67 positive tumor cells (Table 1), while no significant association was observed with other parameters like sex, age, total lymph node dissection, pN Stage, differentiation, surgical procedure, and location. In addition, we investigated the correlation between SENP1 expression and overall patient survival. In a 5-year follow-up cohort, the expression of SENP1 in ESCC patients was inversely associated with their overall survival (Fig. 6E). Kaplan-Meier survival curves indicated that SENP1 expression was a prognostic factor for overall survival in human ESCC (*P* = 0.0297). To verify that SENP1 could be a predictor of long-term prognosis in ESCC patients, we used univariate COX regression analysis to validate the patients’ age, gender, lymph node metastasis rate, pathological T-stage, pathological N-stage, tumor differentiation, and SENP1 expression, respectively. SENP1 was found to be associated with and recurrence-free survival, respectively (Fig. 6F, G). Patients with high SENP1 expression had low overall survival and higher risk of death, and patients with high SENP1 expression had low recurrence-free survival. These results indicate that SENP1 is associated with esophageal carcinogenesis and predicts poor prognosis in patients with ESCC.

## Discussion

Our study has revealed intriguing findings regarding the role of SENP1 in the development of ESCC. Our analysis demonstrated that SENP1 is highly expressed in both human and murine ESCC tissues, suggesting that it plays a crucial role in promoting the onset of ESCC. Using knockdown or condition knockout experiments, we found that suppressing SENP1 can significantly inhibit ESCC growth both in vivo and in vitro. Furthermore, we observed that the deficiency of SENP1 leads to the accumulation of SUMOylation of SIRT6, which further attenuates the cell cycle and cell growth. Finally, our study underscores the association between SENP1 and esophageal carcinogenesis and predicts a poor prognosis in patients with ESCC. Our research represents the first demonstration that specific repression of SENP1 can be an effective therapeutic approach to inhibit ESCC growth.

Interestingly, upon investigating the expression level of SENP1 in patients diagnosed with ESCC, our research has discovered a significant increase in SENP1 expression in ESCC specimens compared to adjacent tissues. This discovery is consistent with the results obtained from the RNA sequencing of mice. One study also showed a correlation between SENP1 expression and the clinicopathological characteristics of tumor patients [32]. We found that elevated SENP1 in ESCC tissues was associated with larger tumor size and advanced clinical stage in ESCC patients. Furthermore, patients with high expression of SENP1 exhibited a shorter overall survival period, implying that SENP1 promotes tumor progression. Through loss-of-function approaches, we further investigated the biological function of SENP1 in ESCC. Our findings revealed that SENP1 enhances the proliferation and migration of ESCC cells.

We discovered that SENP1 regulates the cell proliferation pathway via bulk RNAseq analysis. Our research further revealed that inhibiting SENP1 could result in the downregulation of target genes, including TK1, GMNN, CDK1, and CCNA2 [33–35], leading to cell cycle arrest at the G0/1 phase, which in turn suppresses ESCC development.

Since there’s no report on direct SUMOylation of cyclin genes, we hypothesized that SENP1 might regulate cyclin gene expression via a specific gene pathway. Our previous research has shown that SUMOylation plays a crucial role in modulating deacetylation of SIRT6, which is essential for its tumor suppressive activity [31]. SIRT6 is a tumor suppressor that inhibits cell proliferation and tumorigenesis by partially repressing c-MYC-dependent genes involved in ribosome biogenesis [9]. As SIRT6 is specifically modified by SUMOylation to repress c-MYC target genes, we speculate that SENP1 can influence ESCC progression by deSUMOylating SIRT6. Our findings revealed that knocking down SENP1 does not impact the expression of SIRT6. Nonetheless, deleting SIRT6 impedes the function of SENP1 knockdown, leading to the up-regulation of the expression of pro-proliferative proteins, indicating the involvement of SIRT6. We therefore demonstrate that SENP1 de-SUMOylate SIRT6 inhibits its deacetylase activity. SIRT6 was reported to reduce the acetylation of H3K56 (H3K56ac) [36, 37], and we monitored H3K56ac expression in cell lines with shSENP1 and siSIRT6. Our findings suggested that while shSENP1 reduced H3K56ac expression, siSIRT6 counteracted this effect. In KYSE150 cells with simultaneous knockdown of SENP1 and SIRT6, we found that only SIRT6 WT significantly decreased the acetylation level of H3K56, but the deacetylation of H3K56 was almost abolished in SUMOylation-deficient SIRT6 cells (4KR). As a result, SIRT6-4KR promotes the proliferation of esophageal cancer cells compared to SIRT6 WT.

In conclusion, this study sheds light on a potential mechanism that regulates the malignant progression of ESCC through the SENP1-SIRT6 axis. Our findings deepen the understanding of the role of SENP1 in ESCC progression. This could offer a new perspective and a therapeutic target for the prognosis and treatment of ESCC.

## Electronic supplementary material

Below is the link to the electronic supplementary material.

Supplementary Material 1

Supplementary Material 2

Supplementary Material 3

## Data Availability

The data that support the findings of this study are available on request from the corresponding author upon reasonable request.
