# Supplementary figures and images for: Suppressing SENP1 inhibits esophageal squamous carcinoma cell growth via SIRT6 SUMOylation
Source: Cell Oncol (Dordr). 2024 Jul 2;48(1):67–81. doi: 10.1007/s13402-024-00956-4 (PMC11850494; doi:10.1007/s13402-024-00956-4)

A

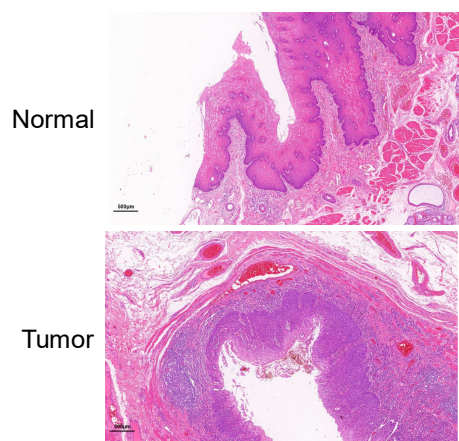

B

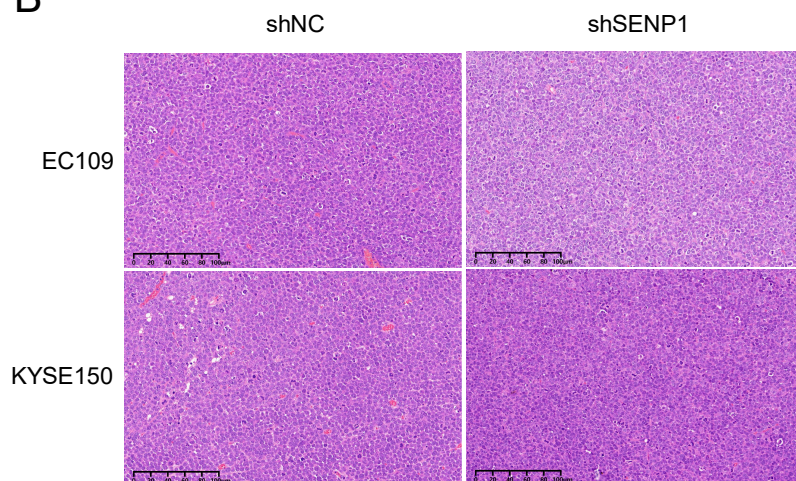

C

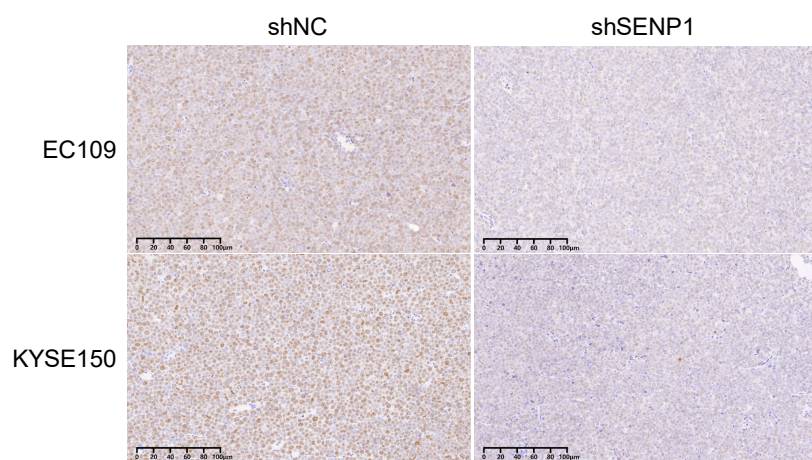

D

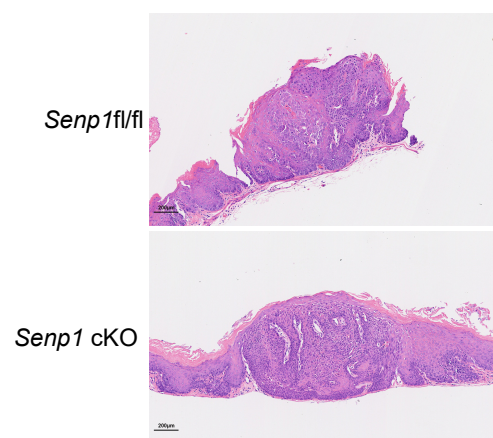

E

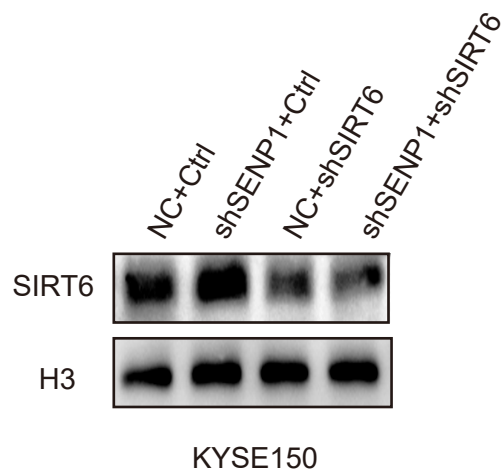

F

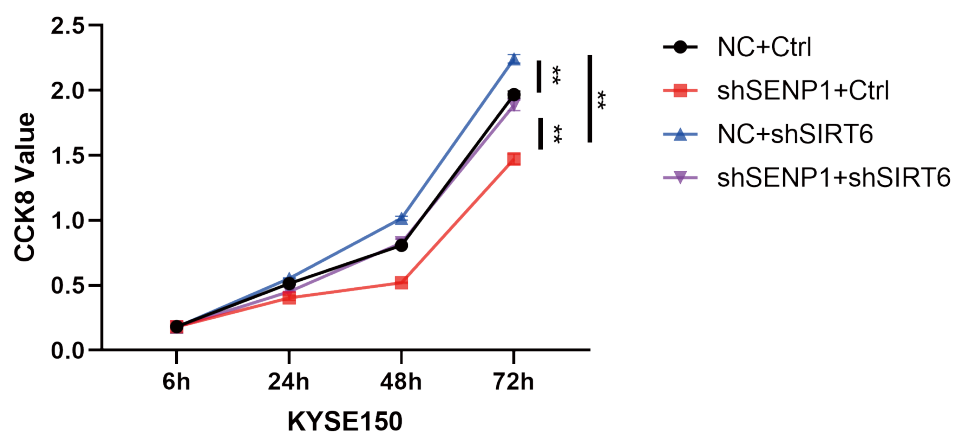

Supplement: Supplementary file 1 — Supplementary Material 1 [file 13402_2024_956_MOESM1_ESM.pdf]
