# Supplementary material for: Suppressing SENP1 inhibits esophageal squamous carcinoma cell growth via SIRT6 SUMOylation
Source: Cell Oncol (Dordr). 2024 Jul 2;48(1):67–81. doi: 10.1007/s13402-024-00956-4 (PMC11850494; doi:10.1007/s13402-024-00956-4)
Supplement: Supplementary file 2 — Supplementary Material 2 [file 13402_2024_956_MOESM2_ESM.docx]

STEP 1: DATA download


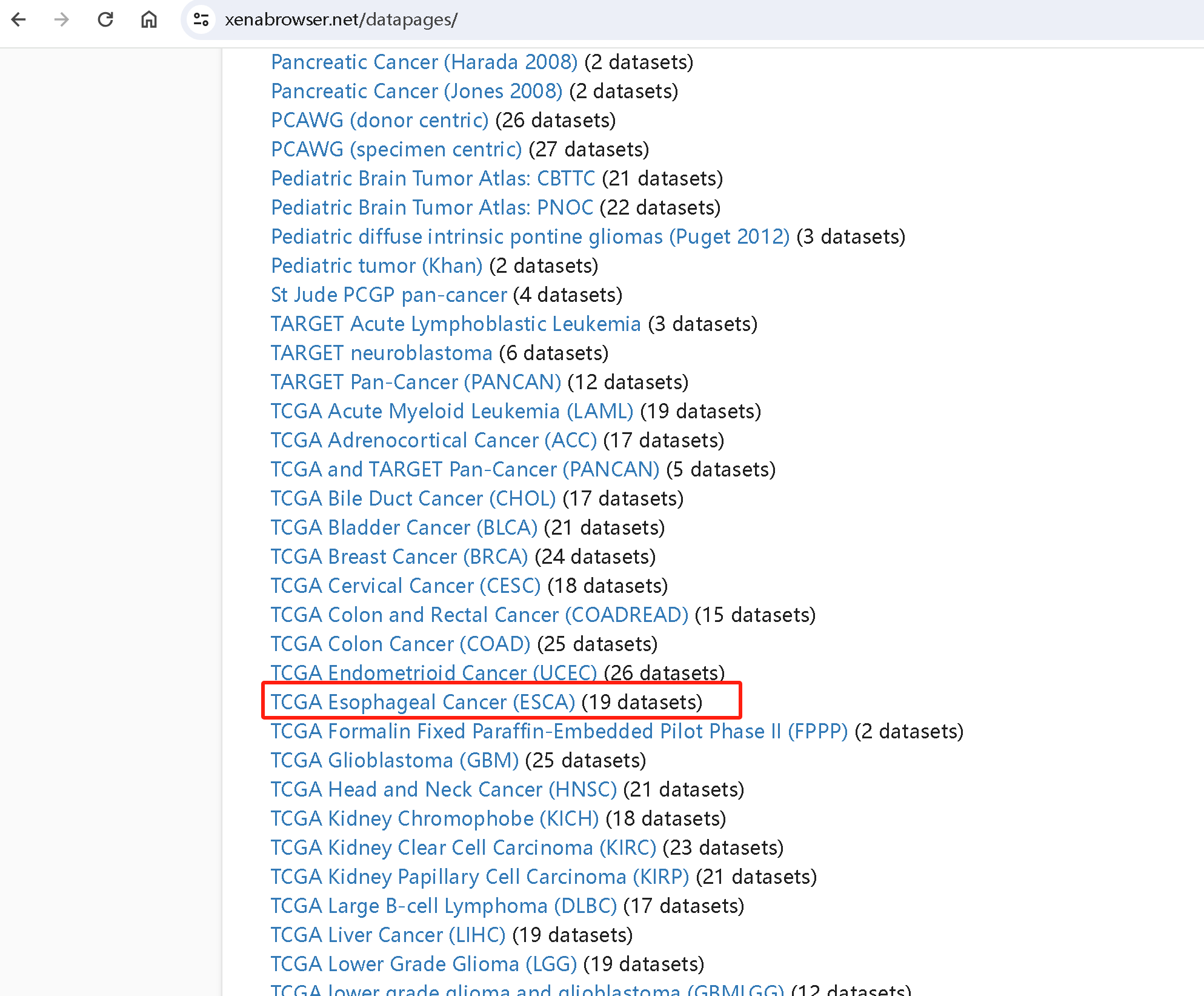


1. Gene Expression


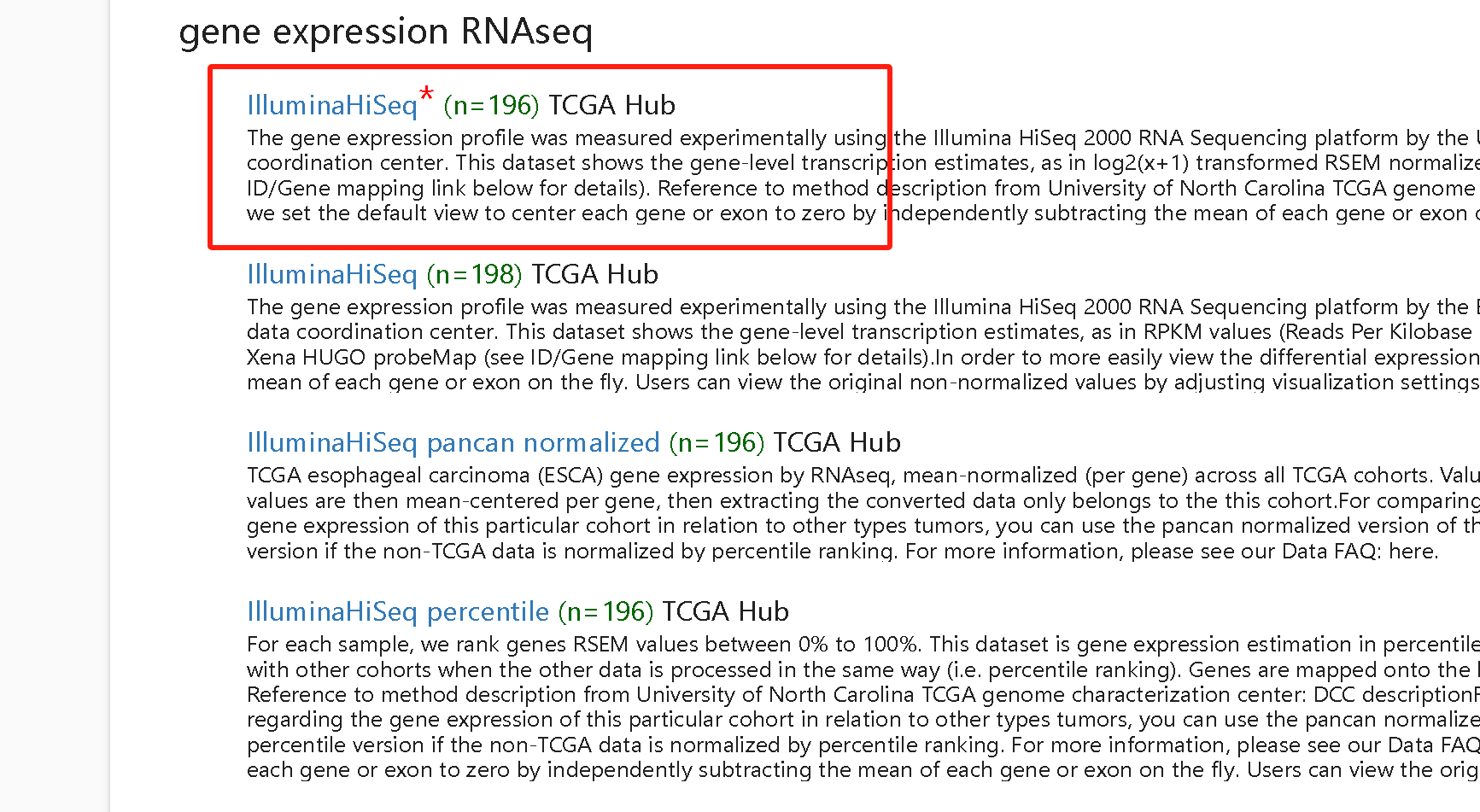


1. Clinical Phenotypes


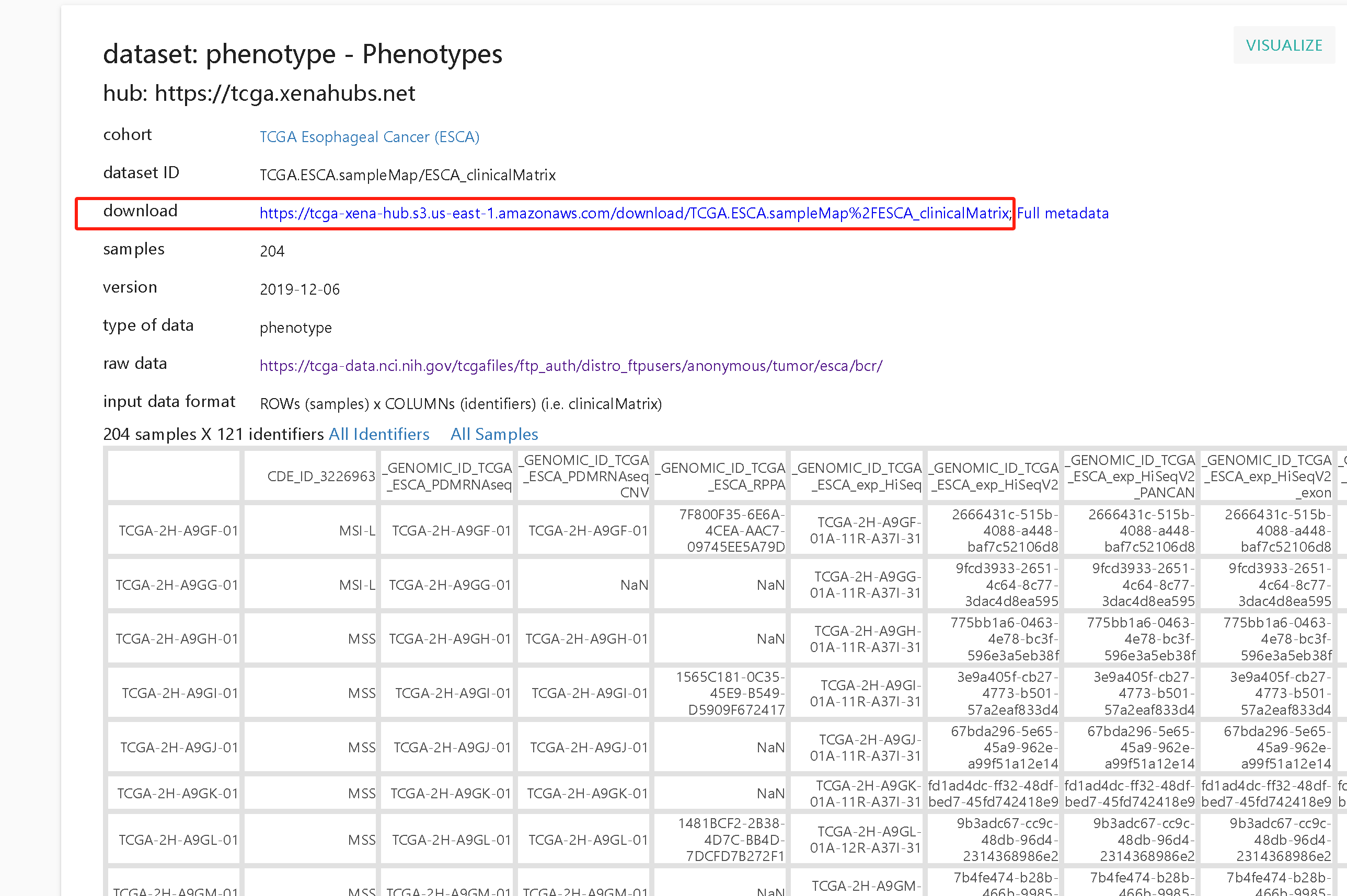


STEP 2 DATA meta

STEP 3 Data processing

According to the sample naming rules of TCGA, the samples ending in -11A are normal tissue samples, so we divided them into squamous cell carcinoma, adenocarcinoma and normal samples according to the sample number and pathological type, and excluded those whose sequencing results were 0.

TCGA-ESCA data is downloaded from UCSC Xena (https://xenabrowser.net/), select the TCGA Esophageal Cancer (ESCA) database, and download phenotype data and gene expression RNAseq data at the same time. We paired the two sets of data to obtain data on ESCC and normal tissue. Finally, we obtained 11 cases of normal tissue samples and 96 cases of ESCC sample data.
